# Direct and Indirect Predictors of Medication Adherence With Bipolar Disorder: Path Analysis

**DOI:** 10.2196/44059

**Published:** 2023-02-07

**Authors:** Bar Cohen, Andrew Sixsmith, Ariel Pollock Star, Ophir Haglili, Norm O'Rourke

**Affiliations:** 1 Goldman Medical School Faculty of Health Sciences Ben-Gurion University of the Negev Be'er Sheva Israel; 2 Science and Technology for Aging Research Institute Simon Fraser University Vancouver, BC Canada; 3 Department of Epidemiology, Biostatistics and Community Health Sciences Ben-Gurion University of the Negev Be'er Sheva Israel; 4 Department of Psychology Faculty of Humanities and Social Sciences Ben-Gurion University of the Negev Be'er Sheva Israel; 5 Center for Multidisciplinary Research in Aging Faculty of Health Sciences Ben-Gurion University of the Negev Be'er Sheva Israel

**Keywords:** alcohol misuse, bipolar disorder, cognitive loss, depression, hypo/mania, mania, medication adherence, mental health, path analysis, perceived cognitive failures, polypharmacy, psychiatric disorder, psychosocial

## Abstract

**Background:**

Despite the efficacy of treatment and severity of symptoms, medication adherence by many with bipolar disorder (BD) is variable at best. This poses a significant challenge for BD care management.

**Objective:**

For this study, we set out to identify psychosocial and psychiatric predictors of medication adherence with BD.

**Methods:**

Using microtargeted social media advertising, we recruited an international sample of young and older adults with BD living in North America (Canada and the United States), Western Europe (eg, United Kingdom and Ireland), Australia and New Zealand (N=92). On average, participants were 55.35 (SD 9.65; range 22-73) years of age, had been diagnosed with BD 14.25 (SD 11.14; range 1-46) years ago, and were currently prescribed 2.40 (SD 1.28; range 0-6) psychotropic medications. Participants completed questionnaires online including the Morisky Medication Adherence Scale.

**Results:**

Medication adherence did not significantly differ across BD subtypes, country of residence, or prescription of lithium versus other mood stabilizers (eg, anticonvulsants). Path analyses indicate that alcohol misuse and subjective or perceived cognitive failures are direct predictors of medication adherence. BD symptoms, psychological well-being, and the number of comorbid psychiatric conditions emerged as indirect predictors of medication adherence via perceived cognitive failures.

**Conclusions:**

Alcohol misuse did not predict perceived cognitive failures. Nor did age predict medication adherence or cognitive failures. This is noteworthy given the 51-year age range of participants. That is, persons in their 20s with BD reported similar levels of medication adherence and perceived cognitive failures as those in their 60s. This suggests that perceived cognitive loss is a facet of adult life with BD, in contrast to the assumption that accelerated cognitive aging with BD begins in midlife.

## Introduction

### Background

Bipolar disorder (BD) is a chronic mental health condition defined by cycles of depression and mania or hypomania; symptoms are often experienced concurrently [1]. With onset for most in their late teens or early 20s, adults live for many years with BD, euthymic most of the time or with subclinical symptoms.

Pharmacotherapy is the first line of treatment for BD [2,3], generally beginning with a mood stabilizer (eg, lithium or anticonvulsants). Antidepressants, antipsychotics, anxiolytics, and hypnotics are also commonly prescribed to treat both BD and comorbid psychiatric conditions (eg, anxiety). Thus, polypharmacy with BD is the norm. Given the chronic nature of BD and the importance of treatment [4] to quality of life [5], understanding factors associated with medication adherence is integral to clinical management and long-term well-being.

### Medication Adherence

Across mental health conditions, various common factors affect medication adherence, including the number and type of medications prescribed. Clinicians generally strive for monotherapy, as a single medication enables better adherence compared to multidrug regimens; the more drugs prescribed, the lower the medication adherence [6]. Also, drugs taken multiple times a day have lower adherence compared to those taken once a day or less [6]. Number and severity of side effects also reduce medication adherence [2,7]. Germane to BD, cognitive decline is associated with lower adherence due to various factors (eg, forgetfulness and misplacing medication).

Medication adherence is closely associated with the symptoms of the underlying condition as adherence both affects, and is affected by, symptoms. With physical health conditions, adherence increases with the onset of symptoms. With mental health conditions, however, associations between symptoms and adherence are more complex. With BD, the range and contrary effects of symptoms further affect adherence. During manic episodes, for instance, patients can experience euphoria and omnipotence, and do not see themselves as unwell; on the contrary, this *anosognosia*, or lack of symptom insight, predicts poor medication adherence. While euthymic or while experiencing psychosis or manic symptoms, persons with BD may consciously discontinue pharmacotherapy [8,9].

Existing research suggests that predictors of poor medication adherence with BD include severity and type of symptoms (both depression and hypo/mania; hypomania + mania = hypo/mania: a continuum where the point of transition is not always apparent), various sociodemographic factors (eg, education), substance misuse, medication regimen, and therapeutic relationship [10-13]. Generally, persons with BD report relatively low medication adherence [14]; in fact, adherence is poor for 20% to 60% of adults with BD [12]. This is problematic for clinical management of a chronic mental health condition with severe symptoms (eg, self-harm) [15] and effective pharmacotherapy [16].

For this study, we set out to identify both direct and indirect predictors of medication adherence with BD. Multivariate analyses included both psychiatric and psychosocial factors. Medication adherence was also compared across BD subtypes, country of residence, and prescription of lithium versus other mood stabilizers.

## Methods

### Ethics Approval

Ethics approval for the BADAS (Bipolar Affective Disorder and older Adults) study was received from the Human Ethics Research Committee at Simon Fraser University (2014s0375).

### Recruitment and Data Collection

For the BADAS study, an international sample of young and older adults with BD was recruited using microtargeted Facebook advertising [17-19]. Recruitment notices were sent only to those with “BD interests” (a euphemism used by Facebook to avoid diagnostic labels). Machine-generated algorithms calculated by social media platforms are distinctive not so much for their sensitivity but for their specificity (ie, exclusion of those who do not have BD). That is, participants recruited via social media are not representative of the population; in fact, they may be more symptomatic than BD outpatients attending mood disorder clinics [20], yet we can be confident that the participants were persons with BD, as only Facebook users with BD received recruitment notices [21]. In previous research in which participants with BD were recruited via Facebook, diagnoses were confirmed in clinical interviews [22,23] and corroborated by cohabiting partners (ie, proxy informants) [18].

By clicking on advertisements appearing within Facebook newsfeeds or sidebar, participants were directed to an online form that specified study inclusion criteria [21]. Thereafter, they completed counterbalanced questionnaires on a secure server; responses were encrypted before transmission [24].

Participants completed 2 sets of online questionnaires an average of 67 (SD 18) days apart. BD symptoms were measured at both points of measurement. In the second wave, we also measured BD medication, adherence, and current symptom levels (both depression and hypo/mania).

Most participants (N=92) indicated that they were currently prescribed 1 or more mood stabilizer (n=54, 59%) and ≥1 antidepressant (n=59, 64%); smaller numbers listed 1 or more anxiolytic (n=36, 39%) and 1 or more antipsychotic (n=35, 38%). By category, lithium (n=78), bupropion (n=43), clonazepam (n=55), and quetiapine (n=68) were the medications most listed by participants (ie, mood stabilizer, antidepressant, anxiolytic, and antipsychotic, respectively).

Participants were also asked to categorize each of these medications: 96% (88/92) correctly specified and categorized both mood stabilizers and anxiolytics, 97% (89/92) for antidepressants, and 86% (79/92) for antipsychotics. This high level of accuracy fosters confidence that participants were, in fact, persons with BD, who were informed about their condition and treatment.

### Instruments

The 8-item Morisky Medication Adherence Scale (MMAS-8) is a self-report scale widely used across settings and clinical populations [25]. Factors known to be associated with MMAS-8 responses by BD patients include depressive symptoms, socioeconomic status, family support, alcohol consumption [26], and belief in the efficacy of treatment [27]. Test-retest reliability over 2 weeks has been reported as *r*=0.72 [28]. MMAS-8 responses strongly correlate with the Medication Adherence Report Scale (*r*=0.56), suggesting concurrent validity [29].

The Bipolar Disorder Symptom Scale (BDS_x_) [30,31] was developed to measure symptoms of both depression and hypo/mania. Respondents indicate the degree to which 20 mood adjectives correspond to how they feel at that moment. Responses are provided on a Likert scale ranging from *not at all* (0) to *a lot* (2). Elevated responses to both depression and hypo/mania subscales correspond to blind psychiatric diagnoses of depressive and hypo/manic mood episodes [1].

The construct validity of the BDS_x_ is supported by quality of life with BD [20], across BD subtypes [32], and by responses from cohabiting partners [18]. For instance, affrontive symptoms of hypo/mania appear to have the greatest negative impact on partners, especially when couples are physically together (GPS coordinates) [18].

The Alcohol Use Disorders Identification Test (AUDIT) [33] is a 10-item self-report questionnaire measuring alcohol consumption. The internal consistency of AUDIT responses is within acceptable parameters (.74<α<.96). Concurrent validity has been demonstrated relative to the Michigan Alcoholism Screening Test (*r*=0.77) [34]. Research using the AUDIT suggests that cognitive loss is greater for BD patients with concomitant alcohol use disorders [35].

The 17-item Cognitive Failures Questionnaire (CFQ) [36] measures subjective or perceived failures in perception, memory, and motor function. CFQ responses correlate positively with accident proneness, human error, and psychological strain, and negatively with executive functioning [37]. CFQ responses have been validated in general samples [38] and older adults with BD [20]. CFQ responses might be an early indicator of neurodegeneration perceived by patients before objective measurement [39].

The Spiritual Index of Well-Being (SiWB) was developed for health-related quality of life research with various clinical populations [40]. Test-retest reliability has been reported as *r*=0.78 over 2 weeks. Good internal consistency has been reported with various adult populations (.84<α<.90) [40].

The SiWB does not measure religiosity, but self-efficacy (α=.86) and life scheme (α=.86) [41]. In essence, the SiWB is a measure of psychological well-being. Responses to the SiWB are strongly correlated with self-transcendence (*r*=0.59), supporting the construct validity of the scale [41]. Among older adults, SiWB responses strongly correlate with both optimism (*r*=0.51) and meaning in life (*r*=0.46) [42]. SiWB responses are inversely correlated with depression in primary care [43]. Spirituality and religiosity appear related to quality of life over time with BD [44].

A sociodemographic questionnaire was constructed to collect descriptive and mental health information. Participants indicated their country of residence (from a drop-down menu); number of years of education; work or occupation; current employment; and relationship status. At recruitment, they were asked if they had BD and subtype, if known; at time 2, they were asked if they had been diagnosed with BD by a clinician (eg, psychiatrist) and date of BD diagnosis (month and year). We included only those who indicated that they both had BD and had been diagnosed with BD.

### Statistical Methods

Path analysis was performed for this study as a three-step process [45]. A hypothesized model was first tested in which all independent variables were assumed to directly predict medication adherence, nonsignificant paths were deleted, and statistically significant paths not initially hypothesized were added if supported by existing research or theory.

Path analysis is an extension of linear regression with three significant advantages [45]. Path analysis allows us to simultaneously predict one or more dependent variables (touched by arrowheads in path models). Arrows pointing from independent to dependent variables represent significant prediction (ie, critical ratio [CR] values >|1.96|, *P*<.05). Path analysis is a multivariate statistical procedure, meaning that all significant paths emerge concurrently (ie, over and above other statistically significant results).

Path models allow us to identify both direct and indirect predictors of medication adherence [45]. Indirect prediction occurs via other variables (ie, ≥2 pathways between variables). In complex or more nuanced path models, variables can have direct and indirect effects on dependent variables, and indirect effects can be of equal or greater magnitude than direct effects (total effects = direct + indirect effects).

Computing path analyses with structural equation modeling software allows us to obtain goodness of fit information for the overall model. Good model fit is required to interpret path models [46]. In accord with convention, we report three goodness-of-fit-indices to assess overall model fit of path models to data: an incremental (comparative fit index [CFI]), an absolute (standardized root mean residual [SRMR]), and a parsimonious (root mean square error of approximation [RMSEA]) fit index. Ideal SRMR and RMSEA values are less than 0.055, whereas ideal CFI values are greater than 0.95 [45].

Descriptive and comparative analyses were performed using SPSS (version 28; IBM Corp) and path analyses were performed using AMOS (version 26; IBM Corp).

## Results

### Descriptive Features

For this study, 92 BADAS participants were identified who reported medication information and medication adherence (mean age 55.35, SD 9.65 years). Their age ranged from 22 to 73 years; there were 35 men and 57 women. On average, participants had been diagnosed with BD 14.25 (SD 11.14) years ago. They were currently prescribed an average of 2.40 (SD 1.28; range 0-6) psychotropic medications. Descriptive statistics for study variables are reported in Table 1.

**Table 1:** Descriptive statistics and study variables (N=92).

		Men, mean (SD)	Women, mean (SD)	α	*t* (*df*)	*P* value
Age (years)		56.48 (9.33)	54.94 (9.86)	^—a^	0.72 (85)	.47
Education (years)		11.33 (6.06)	11.06 (5.77)	—	0.21 (84)	.83
Duration of bipolar disorder diagnosis (years)		13.78 (12.40)	14.59 (10.44)	—	–0.30 (81)	.77
Comorbid psychiatric conditions, n		0.73 (1.10)	0.96 (1.21)	—	–0.91 (85)	.37
Total psychotropic prescriptions, n		2.18 (1.24)	2.48 (1.34)	—	–1.01 (85)	.30
Morisky Medication Adherence Scale (score)		7.22 (1.61)	6.41 (2.81)	.70	–1.74 (85)	.09
Bipolar Disorder Symptom Scale (BDS_x_; score)						
	Depression	8.06 (5.20)	10.17 (5.79)	.91	–1.71 (85)	.09
	Hypo/mania	4.27 (3.81)	4.83 (4.20)	.83	–0.63 (85)	.53
Spiritual Index of Well-Being (score)		38.88 (12.99)	34.44 (10.65)	.94	1.73 (85)	.10
Alcohol Use Disorders Identification Test (score)		4.91 (4.98)	4.50 (5.65)	.86	0.34 (85)	.73
Cognitive Failures Questionnaire (score)		48.24 (19.51)	56.72 (19.81)	.95	1.95 (85)	.06

^a^Not applicable.

Most participants lived in North America (Canada: n=30, United States: n=30) with the remainder living in the UK or Ireland (n=22), Australia or New Zealand (n=8), or continental Europe (n=2); n=5 did not specify. They had completed an average of 11.16 (SD 5.85; range 1-22) years of education. The plurality worked in managerial/professional (n=25) or administrative/clerical positions (n=22); 21 were currently retired or receiving a disability pension. Almost all participants were White (82/92, 89%).

Of those reporting a BD subtype, a plurality specified a BD II diagnosis (n=31), followed by BD not otherwise specified (n=21) and BD I (n=14). Consistent with BD prevalence data [47], few indicated that they had been diagnosed with cyclothymic disorder (n=6). Reported medication adherence did not differ by BD subtype (*F*_3,68_=0.20; *P*=.89), nor did adherence differ between those prescribed lithium carbonate versus other mood stabilizers (eg, valproic acid; t_51_=0.63; *P*=.53). Also of note, adherence did not differ between those concurrently prescribed both a mood stabilizer and an antidepressant versus one or more mood stabilizers alone (ie, no antidepressants; *t*_90_=1.76; *P*=.08).

The relative frequency of BD subtypes did not differ by country of residence (*χ*^2^_6_=3.10; *P*=.80), nor was lithium more widely prescribed in one region than others (*χ*^2^_6_=4.72; *P*=.58). More participants in North America were prescribed one or more antidepressants (50/60, 83%) than those in western Europe (15/22, 68%) or Australia and New Zealand (4/8, 50%), but this difference is not statistically significant (*χ*^2^_2_=5.56; *P*=.06). Medication adherence also did not differ by country (*F*_2,87_=0.32; *P*=.73).

Moreover, medication adherence was not associated with age (*r*=0.13; *P*=.23) or duration of BD diagnosis (*r*=0.10; *P*=.38). A small inverse association emerged between adherence and number of psychotropic medications (*r*=–0.26; *P*=.01).

### Path Analyses

We computed path analyses to identify both direct and indirect predictors of medication adherence with BD. We hypothesized that psychological well-being and BD symptoms (both depression and hypo/mania) would significantly predict medication adherence. The number of psychotropic medications, alcohol misuse, and perceived cognitive failures were also assumed to predict adherence. A sample of 92 participants is small but sufficient to detect medium to large effect sizes with 5 to 6 independent variables, where α=.05 and *d*=.80 [48].

Goodness of fit was within optimal parameters for two of three statistics examined (*χ*^2^_13_=11.17; *P*=.60). The comparative fit index (CFI≥0.95; CFI=0.99) and the root mean square error of approximation (RMSEA≤0.05; RMSEA=0.001) were both within ideal parameters (0<RMSEA 90% confidence limits <0.091); whereas the standardized root mean square residual was adequate (SRMR≤0.080; SRMR=0.075).

A more nuanced model emerged than hypothesized. More precisely, only alcohol misuse and perceived cognitive failures emerged as direct predictors of medication adherence; their relative magnitude is similar (ie, β=–.23 and β=–.28, respectively). Neither symptoms of depression nor hypo/mania emerged as direct predictors of medication adherence; instead, both are predicted by psychosocial factors, which directly and indirectly predict medication adherence. Perceived cognitive failures predict symptoms of depression (β=.47, *P*<.01), hypo/mania (β=.42, *P*<.01), and the absence of psychological well-being (β=–.52, *P*<.01). Number of comorbid psychiatric conditions is also inversely associated with psychological well-being (β=–.24, *P*<.01).

Alcohol misuse predicts medication adherence, not psychological well-being, BD symptoms, or perceived cognitive failures. Age is not associated with cognitive failures (*r*=0.13, *P*=.23). This finding is noteworthy given the 51-year age range of participants (22-73 years). Prior research has identified perceived cognitive failures as significantly associated with quality of life for older adults with BD [14]. The results of this study suggest that perceived cognitive failures are not exclusive to later life with BD.

### Direct and Indirect Effects

**Figure 1:**
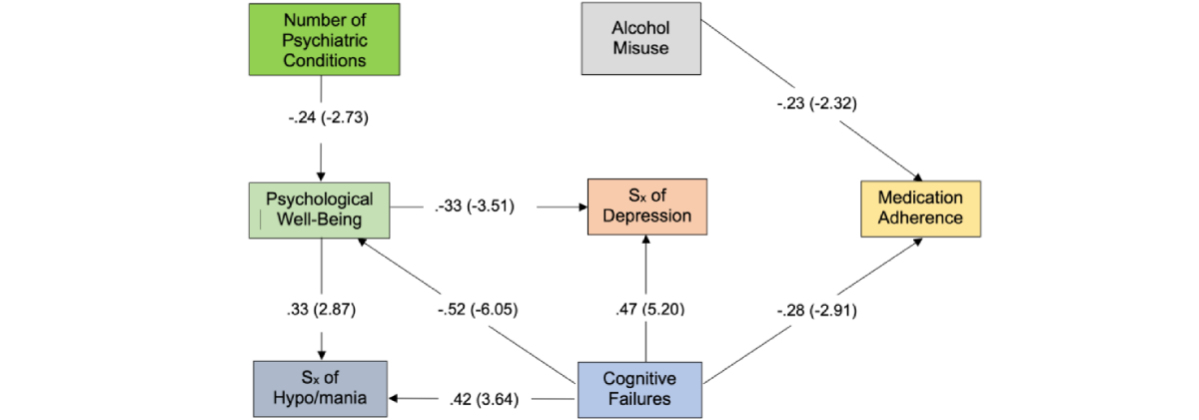
Note: Parameters expressed as maximum likelihood estimates (standardized solution). Parenthetical numbers indicate significance levels (CR > |1.96|, *P*<.05; CR > |2.58|, *P*<.01). Sx: symptoms.

As shown in Figure 1 and Table 2, the number of comorbid psychiatric conditions indirectly predicts both symptoms of depression and hypo/mania via psychological well-being. Also, perceived cognitive failures directly and indirectly predict depression and hypo/mania, again via psychological well-being. Of note, the effect of cognitive failures on hypo/mania is bidirectional. A positive direct effect emerged between perceived cognitive failures and symptoms of hypo/mania, which is negated somewhat by a negative indirect effect via psychological well-being. This positive association between hypo/mania and well-being is contrary to prior research suggesting no association between symptoms of hypo/mania and life satisfaction [49].

**Table 2:** Direct, indirect, and total variance explained.

		Comorbid Conditions	Cognitive failures	Alcohol misuse	Well-being
**Well-being**					
	Direct	–0.24	–0.52		
	Indirect	0	0		
	Total^a^	–0.24	–0.52		
**Medication adherence**					
	Direct		–0.28	–0.23	
	Indirect		0	0	
	Total		–0.28	–0.23	
**Hypo/mania symptoms**					
	Direct	0	0.42		0.33
	Indirect	–0.08	–0.17		0
	Total	–0.08	0.25		0.33
**Depression symptoms**					
	Direct	0	0.47		–0.31
	Indirect	0.07	0.16		0
	Total	0.07	0.63		–0.31

^a^Total values are a sum of direct and indirect values presented in the two immediately preceding rows.

## Discussion

### Principal Findings

For this study, we identified an international sample of young and older adults with BD. Few differences emerged in medication adherence across regions, medications, or BD subtypes. Most participants in North America were prescribed an antidepressant in contrast to other regions, but this difference did not attain statistical significance.

Alcohol misuse and perceived cognitive failures both emerged as significant direct predictors of medication adherence. Number of comorbid psychiatric conditions, psychological well-being, and BD symptoms emerged as indirect predictors via cognitive failures. This finding is in accord with existing research indicating that perceived cognitive failures are significant predictors of quality of life among older adults with BD [20].

BD symptoms, both depression and hypo/mania, are significantly associated with perceived cognitive failures when reported contemporaneously; this effect appears to be both direct and indirect (via psychological well-being). Of note, the direction of direct versus indirect effects differs between cognitive failures and hypo/mania. That is, perceived cognitive failures significantly and directly predict symptoms of hypo/mania, yet the indirect effect via psychological well-being is negative. This suggests that different BD symptoms are associated with certain types of perceived cognitive failures and not others. This bidirectional association underscores the importance of examining both direct and indirect effects in path analyses, not just total variance explained.

These findings are somewhat in accord with previously published research. For instance, certain BD-related factors (ie, number of comorbid psychiatric conditions) emerged as indirectly associated with medication adherence. Our findings corroborate other research demonstrating an inverse relationship between alcohol misuse and medication adherence [10]. However, we found no associations between type of medication regimen and adherence [11]; nor did current BD symptoms, either depression or hypo/mania, directly predict medication adherence [12].

Also noteworthy are factors that appear unrelated to medication adherence, such as medication regimen, duration of diagnosis, and age. The latter finding is noteworthy given the 51-year age range of participants; that is, persons in their 20s with BD reported similar levels of medication adherence and cognitive failures as those in their 60s. This suggests that perceived cognitive loss is a facet of adult life for many with BD, in contrast to the assumption that accelerated cognitive aging with BD begins in midlife [50,51]. Further study is required.

### Limitations and Future Research

A primary limitation of this study is measurement of medication adherence by self-report only. Though the MMAS-8 has been well studied, including BD [52], adherence is complicated to measure, especially with mental health conditions expressed by affective symptoms rather than objective markers. Further measurement of adherence using other methods (eg, pharmacy records) shoould corroborate self-reported responses.

The same is true for self-reported cognitive errors, which are not strongly correlated with performance on neurocognitive test batteries. This has led some to question the validity and utility of self-reported cognitive deficits. It has been suggested that self-reported cognitive deficits are unrelated to well-being with BD [53,54]. This assumption is challenged by an initial BADAS study reporting that perceived cognitive failures directly and indirectly predicted suicide ideation in older adults with BD [20]. Self-reported or perceived cognitive deficits may not reflect neuropsychological test scores, yet more research is needed to better understand associations with adherence.

The results of this study need to be replicated with larger samples and over time. Our sample was sufficient to compute a path model with 6 independent variables; a larger sample may have enabled us to detect small effect sizes and more distal predictors of medication adherence with BD. The direction of cross-sectional associations reported herein may have differed if associations had been measured over time. For instance, poor medication adherence may well predict future symptoms of both depression and hypo/mania; that contemporaneous association was not found. Repeated data collection over an extended interval is needed to more fully understand factors associated with medication adherence with BD. This includes recruitment of participants with BD using more traditional methods (ie, recruitment at mood disorder clinics).

Despite these limitations, we were able to recruit an international sample of young and older adults with BD sufficient for comparative and path analyses. This allowed us to identify both direct and indirect predictors of medication adherence. Our model includes BD symptoms (both depression and hypo/mania), comorbid psychiatric conditions, psychological well-being, and alcohol misuse. When examined contemporaneously, perceived cognitive failures appear most strongly associated with adherence with BD.

### Summary

BD is a chronic mental health condition with onset in early life that persists across adult life. Despite the severity of symptoms and efficacy of pharmacotherapy, medication adherence by many with BD is problematic [12,14]. The results of this study suggest that cognitive failures and alcohol misuse are direct predictors of poor adherence. BD symptoms appear to affect medication adherence via bidirectional effects upon other variables.

## Abbreviations

AUDIT: Alcohol Use Disorders Identification Test
BADAS: Bipolar Affective Disorders and older Adults Study
BD: Bipolar disorder
BDSx: Bipolar Disorder Symptom Scale
CFI: comparative fit index
CFQ: Cognitive Failures Questionnaire
CR: critical ratio
MMAS-8: Morisky Medication Adherence Scale
RMSEA: root mean square error of approximation
SiWB: Spiritual Index of Well-Being
SRMR: standardized root mean residual
